# Simultaneous Two-Vessel Subacute Stent Thrombosis Caused by Clopidogrel Resistance from CYP2C19 Polymorphism

**DOI:** 10.1155/2016/2312078

**Published:** 2016-07-31

**Authors:** Ashwad Afzal, Bimal Patel, Neel Patel, Sudhakar Sattur, Vinod Patel

**Affiliations:** ^1^Department of Internal Medicine and Division of Cardiology, New York Methodist Hospital, Brooklyn, NY 11215, USA; ^2^Surat Municipal Institute of Medical Education and Research, Umarwada, Surat, Gujarat 395010, India; ^3^Department of Cardiology, Guthrie Clinic, Sayre, PA 18840, USA

## Abstract

Clopidogrel resistance from CYP2C19 polymorphism has been associated with stent thrombosis in patients undergoing percutaneous coronary intervention with drug-eluting stents. We present a case of a 76-year-old male who received drug-eluting stents to the right coronary artery and left anterior descending artery for non-ST elevation myocardial infarction and was discharged on dual antiplatelet therapy with aspirin and clopidogrel. He subsequently presented with chest pain from anterior, anteroseptal, and inferior ST segment elevation myocardial infarction. An emergent coronary angiogram revealed acute stent thrombosis with 100% occlusion of RCA and LAD that was successfully treated with thrombus aspiration and angioplasty. Although he was compliant with his dual antiplatelet therapy, he developed stent thrombosis, which was confirmed as clopidogrel resistance from homozygous CYP2C19 polymorphism.

## 1. Introduction

Clopidogrel is recommended as part of a dual antiplatelet strategy in patients with Acute Coronary Syndrome (ACS) who receive percutaneous coronary intervention (PCI) with drug-eluting stent (DES) as it has been shown to decrease the incidence of stent thrombosis [1, 2]. Clopidogrel is a prodrug that requires biotransformation by several hepatic cytochrome P-450 (CYP) isoenzymes including CYP2C19 to become an active metabolite [3]. However, genetic polymorphism of CYP2C19 has been associated with poor clopidogrel metabolism, in turn leading to stent thrombosis and other major adverse cardiovascular events [4].

We present a unique case report of two-vessel subacute stent thrombosis in a patient receiving clopidogrel as part of dual antiplatelet therapy after PCI with DES. This was likely due to clopidogrel resistance despite being compliant with dual antiplatelet therapy. In this report we elaborate on the details of the case and discuss diagnosis and management of patients with clopidogrel resistance.

## 2. Case Presentation

A 76-year-old male with past medical history of hypertension, hyperlipidemia, and diabetes mellitus type 2 was admitted one week priorly with chest pain due to non-ST segment elevation myocardial infarction (NSTEMI). An urgent coronary angiography showed 99% stenosis of the proximal left anterior descending artery (LAD) and 95% stenosis of middle right coronary artery (RCA). The patient underwent PCI with DES. A 2.5 mm × 38 mm Promus DES was placed in the proximal LAD. Three Promus DES were placed in the mid RCA: 2.5 mm × 20 mm, 2.5 mm × 28 mm, and 2.5 mm × 24 mm stent. His peak CK-MB and troponin were 77.4 and 107 ng/L, respectively. The patient was discharged on optimal medical therapy including dual antiplatelet therapy of aspirin and clopidogrel. Due to financial constraints, ticagrelor or prasugrel could not be prescribed. He was seen one week after discharge with no complaints and was compliant with his medications.

**Figure 1:**
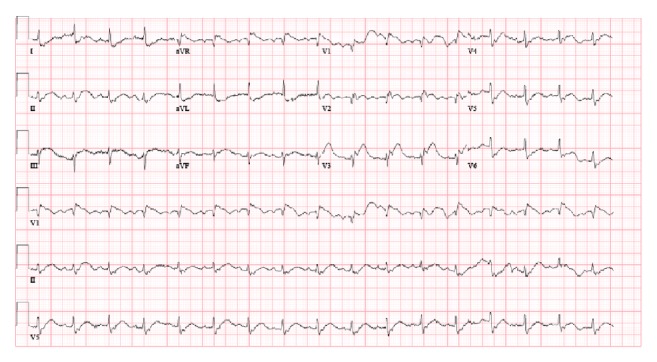
Electrocardiogram on admission showing ST segment elevation in inferior and anteroseptal distribution (STEMI from in-stent thrombosis).

**Figure 2:**
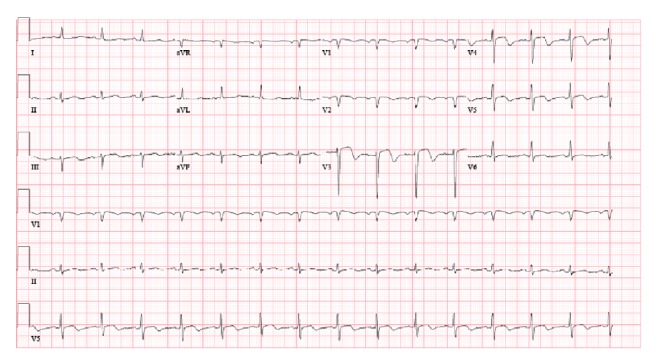
Electrocardiogram after thrombus aspiration and balloon angioplasty showing improvement of ST segment elevation in inferior and anteroseptal leads and ST segment depression in lateral leads.

**Figure 3:**
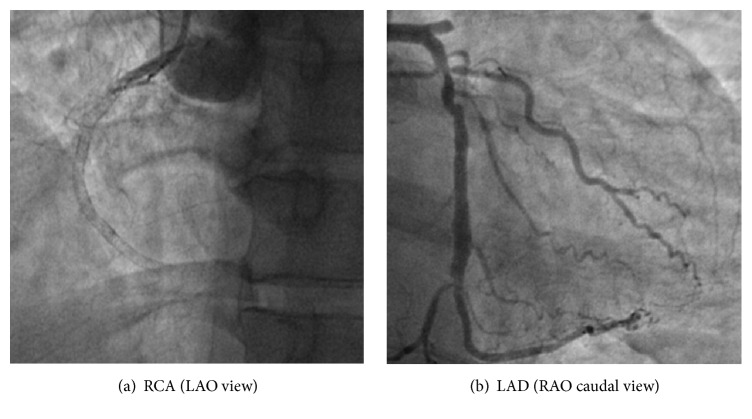
Angiogram revealing in-stent thrombosis of the RCA and LAD.

**Figure 4:**
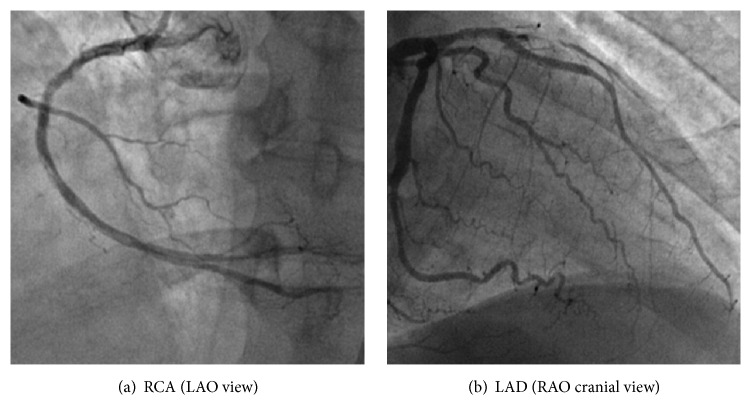
Angiogram after thrombus aspiration and balloon angioplasty of the RCA and LAD.

The patient presented eight days after the index hospitalization with severe, crushing, substernal chest pressure. The electrocardiogram showed ST segment elevation in the inferior and anteroseptal leads (Figure 1). An emergent coronary angiogram was performed revealing 100% occlusion of the proximal LAD and mid RCA due to in-stent thrombosis (Figures 3(a) and 3(b)). The patient also developed sustained ventricular tachycardia prior to PCI that was terminated with external defibrillation. Successful PCI with aspiration thrombectomy and balloon angioplasty was performed using a 2.5 mm × 15 mm NC quantum Apex balloon to the proximal LAD and middle RCA lesion (Figures 4(a) and 4(b)). The patient also developed multiple episodes of symptomatic sinus bradycardia that resolved with atropine and placement of a temporary transvenous pacemaker. We suspected clopidogrel resistance; hence the patient was treated with aspirin and ticagrelor therapy. Further laboratory testing confirmed that patient had homozygous polymorphism of CYP2C19^*∗*^2. This is an established cause of poor clopidogrel metabolism leading to reduced levels of active clopidogrel metabolite. This was the likely cause of two-vessel stent thrombosis leading to STEMI. The patient was monitored in the cardiac intensive care unit with no further events and there was improvement in ST segment elevation on repeat electrocardiogram (Figure 2). A transthoracic echocardiogram showed severe diffuse hypokinesis with abnormal left ventricular relaxation, an ejection fraction of 20%, and no significant valvulopathies. His peak CK-MB and troponin were 113 and 23.9 ng/L, respectively. He was recovering well without any further cardiac events at one-month and three-month clinic follow-up.

## 3. Discussion

Clopidogrel is a prodrug that requires activation by the hepatic cytochrome P-450 system to become an active metabolite [3]. This active metabolite inhibits platelet aggregation by irreversibly binding to platelet ADP receptor P2Y12. Genetic polymorphism of the cytochrome P-450 isoenzymes can affect clopidogrel metabolism. Polymorphism with loss-of-function alleles CYP2C19^*∗*^2 (681G>A) is associated with poor clopidogrel metabolism that is seen in both heterozygous and homozygous patients [5–7]. This defect leads to a mutation in exon 5 that creates an aberrant splice site resulting in a truncated, nonfunctional protein [8]. Mega et al. showed that carriers of at least one CYP2C19 reduced-function allele had a 32.4% relative reduction in active clopidogrel metabolite in comparison to noncarriers (*P* < 0.001) [4]. A low circulating active clopidogrel metabolite causes a diminished platelet response to treatment leading to higher rates of major adverse cardiovascular events [4, 9]. Genotype analysis in this patient showed homozygous loss-of-function alleles of CYP2C19^*∗*^2/^*∗*^2. This led to inadequate platelet inhibition resulting in two-vessel stent thrombosis. In patients with heterozygous CYP2C19^*∗*^2 alleles, higher doses of clopidogrel have been shown to overcome the genetic resistance and improve the platelet inhibition [10]. However, higher doses of clopidogrel cannot overcome the resistance and are not an option for CYP2C19^*∗*^2 homozygote patients. An alternative antiplatelet therapy with ticagrelor was used. Ticagrelor is a reversible inhibitor of ADP receptor P2Y12 that has been shown to have a greater and more consistent inhibition of platelet aggregation in comparison to standard clopidogrel dose [11]. It has been shown to reduce the rate of death from myocardial infarction compared to clopidogrel without a significant increase in major bleeding [12].
